# Transferring an ICU Patient at the End of His Life for the Purpose of Organ Donation: Could It Be Considered?

**DOI:** 10.3389/ti.2022.10549

**Published:** 2022-06-22

**Authors:** Matthieu Le Dorze, Bénédicte Gaillard Le Roux, Gérard Audibert, Régis Quéré, Laurent Muller, Sylvain Lavoué, Jean-Christophe Venhard, Pierre-François Perrigault, Olivier Lesieur

**Affiliations:** ^1^ Ethics Committee of the French Society of Anesthesia and Critical Care Medicine (SFAR), Paris, France; ^2^ Department of Anesthesia and Critical Care Medicine, Hôpital Lariboisière AP-HP, Paris, France; ^3^ Université Paris-Saclay, UVSQ, INSERM, CESP, U1018, Villejuif, France; ^4^ Ethics Commission of the French Intensive Care Society (SRLF), Paris, France; ^5^ Pediatric Intensive Care Unit, University Hospital, Nantes, France; ^6^ Department of Anesthesia and Critical Care Medicine, Nancy University Hospital, University of Lorraine, Nancy, France; ^7^ Organ Procurement Organization, Necker University Hospital, Paris, France; ^8^ Department of Anesthesia and Critical Care Medicine, University Hospital, Nîmes, France; ^9^ Intensive Care Unit, University Hospital, Rennes, France; ^10^ Department of Anesthesia and Critical Care Medicine, French Society of Organ Procurement Medicine, University Hospital, Tours, France; ^11^ Department of Anesthesia and Critical Care Medicine, University Hospital, Montpellier, France; ^12^ Intensive Care Unit, General Hospital, La Rochelle, France

**Keywords:** organ donation, end-of-life care, controlled donation after circulatory death, withdrawal of life-sustaining therapies, intensive care units

Dear Editors,

Controlled donation after circulatory death (cDCD) refers to organ donation from patients whose death is defined by circulatory criteria after the planned withdrawal of life-sustaining treatments (WLST) in intensive care units (ICUs) [1]. The development of this type of donation has varied from country to country due to their different legal, ethical, and organizational frameworks, which explain diverse activity levels and transplant outcomes [2]. France began its cDCD program in 2015 with ethical and technical aspects leading to a nationwide protocol. The underlying principle is that the decision to withdraw LST must be made in the patient’s best interest, independently from any consideration regarding organ donation, and that cDCD must not alter end-of-life care [3]. The challenge is not only to identify potential cDCD donors, but also to provide support to grieving families and to give caregivers a reassuring ethical framework [4]. Yet, caregivers can feel particularly uncomfortable when, in practice, end-of-life care and organ donation overlap.

Today, the scarcity of donor organs and the good transplantation outcomes [5, 6] legitimately support the development of cDCD in a context where WLST decisions occurs more and more frequently in ICUs worldwide [7]. This development is limited by technical and organizational aspects, in particular related to the systematic use of normothermic regional perfusion (NRP), which requires technology (NRP device) and expertise (NRP settings and vessels cannulation) not available in all hospitals. It is also likely limited by many ethical issues as cDCD reshapes end-of-life care by introducing the issue of organ donation before the time of death. Thus, cDCD may potentially affect not only the WLST decision-making process but also other end-of-life care practices, such as sedative practices, and the acceptance by relatives and caregivers [8, 9].

For the further development of cDCD, more hospitals should have the technical and organizational capacity to achieve regulatory approval for cDCD. However, beyond ethical issues, this may be limited by technical or organizational aspects, and/or by the relatively small number of potential cDCD donors, and subsequent procedure failure risks. The question is: which ways could be considered to allow cDCD for an ICU end-of-life patient hospitalized in an institution that does not have the resources for cDCD? The first strategy is the use of mobile normothermic regional perfusion and should be preferred as ethical issues are limited in this scenario [10]. When this is not possible, another strategy could be to allow the transfer of an ICU end-of-life patient for the purpose of organ donation to another hospital allowed for cDCD.

The transfer of an ICU end-of-life patient for the purpose of organ donation raises many ethical issues as its potential impact on the patient himself, on his/her relatives and on ICU caregivers. There are many risks, including: not complying with the wishes of a patient unable to express himself; considering the patient from a purely utilitarian perspective; changing end-of-life practices so that the death occurs with a timeframe that allows organ donation; transferring the patient under the presumption of consent, even though the patient is on the registry of refusals (which is only consultable after death in France); affecting experiences and perceptions of relatives through geographic and/or relational discontinuity; having an impact on the experience of caregivers and their motivation to be involved in organ donation.

Overall, in contexts of potential organ donation, end-of life support must always be preserved. The transfer of an ICU end-of-life patient for the purpose of organ donation should remain an exception. Moreover, this exception may only be justified by the aim of complying with the clearly expressed wish of the patient to donate his/her organs after-death. A sole presumption of consent (as stated by French law for conventional organ donation procedures) may not be sufficient. The individual’s values and preferences regarding end-of-life and organ donation must be respected over any utilitarian considerations. This is a key issue while France has adopted the opt-out system. Concerning relatives, they must be clearly informed at each stage of this complex process. Support for them must always be provided. Facilities for transport and accommodation must be offered to them, as well as the return of the body must be mandatory after death and organ procurement. Concerning caregivers, the principle of separation between WLST decisions and organ donation possibility must strictly be respected, end-of-life practices must be applied as they are formalized independently from the possibility of organ donation. The training and support of caregivers involved is a central goal.

Finally, it could be possible if the following four conditions are met:

(1) Arrangements that favor the proximity of the patient to his/her relatives and ICU caregivers, such as the use of a mobile NRP, cannot be implemented locally for technical or organizational reasons.
(2) The clearly expressed wish of the patient to donate organs after death (first-person or relatives testimony, living will, advance directives).
(3) The adherence to a formalized procedure described in Figure 1 that involves the end-of-life patients, the relatives, the ICU team 1 working in the hospital 1, the ICU team 2 working in the hospital 2 allowed for cDCD, and the organ procurement organization. Sharing the details of the case should ensure that ICU team 2 adheres a priori to the decision of ICI team 1 to withdraw LST. Particular attention must be paid to the quality of communication between the two ICU teams, the organ procurement organization (OPO) team and the relatives.
(4) The transparency of the procedure is ensured.

**FIGURE 1:**
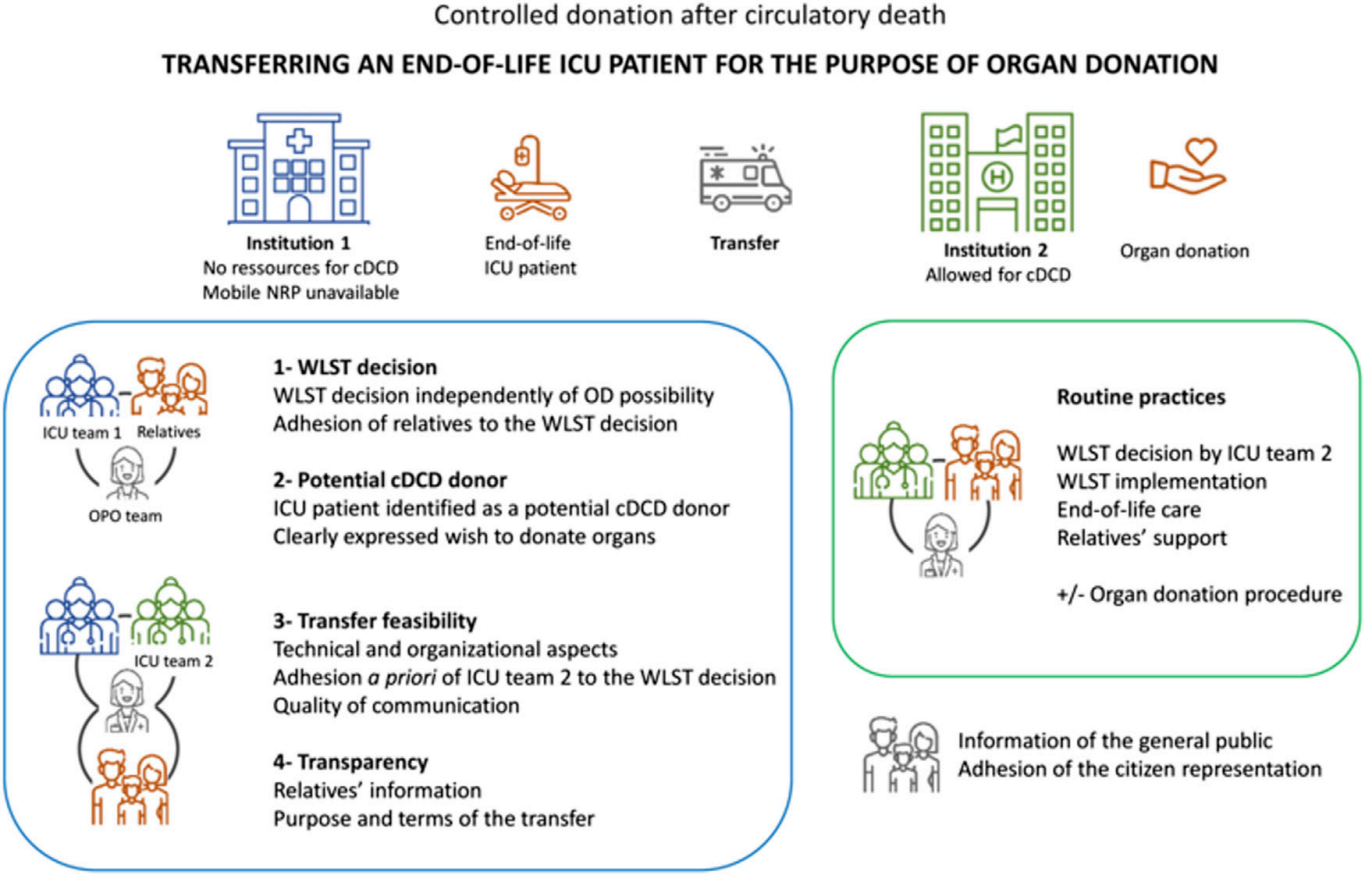
Formalized steps for the transfer of an ICU patient at the end of his/her life for the purpose of organ donation. cDCD, controlled donation after circulatory death; ICU, intensive care units; WLST, withdrawal of life-sustaining treatments; OD, organ donation; OPO, organ procurement organization. Icons made by Monkik, Those Icons, Freepik, kosonicon, Blak1ta, kliwir art, Puckung from www.flaticon.com.

The future development of cDCD needs to address, beyond the technical and organizational aspects, the ethical tension between end-of-life care and organ donation. The future developments of cDCD are ethically reasonable as long as end-of life support is preserved. The information of the general public and the adhesion of the citizen representation to the procedure are crucial and must be pursued.

## Data Availability

The original contributions presented in the study are included in the article/supplementary material, further inquiries can be directed to the corresponding author.

## Abbreviations

cDCD, controlled donation after circulatory death; ICU, intensive care units; NRP, normothermic regional perfusion; WLST, withdrawal of life-sustaining treatments.
